# Supplementary material for: Dynamics of within-host Mycobacterium tuberculosis diversity and heteroresistance during treatment
Source: eBioMedicine. 2020 Apr 28;55:102747. doi: 10.1016/j.ebiom.2020.102747 (PMC7195533; doi:10.1016/j.ebiom.2020.102747)
Supplement: Supplementary file 2 [file mmc2.pdf]

Dynamics of within-host *Mycobacterium tuberculosis* diversity and heteroresistance during treatment

Supplementary Methods 2

Supplementary Tables..5

Supplementary Figures 12

References..19

Supplementary Methods

Supplementary Methods 1: Phenotypic drug susceptibility testing drugs and critical concentrations

Phenotypic drug susceptibility testing (pDST) was performed on Middlebrook 7H11 agar for the following drugs at the specified critical concentrations below. These are based on World Health Organization (WHO) approved critical concentrations where possible(1) but differ in some instances where evidence is limited or for legacy reasons within our laboratory.

**Drug**	**Critical concentration (μg/mL)**	**WHO critical concentration (μg/mL)**
**Rifampicin**	1.0	Same
**Isoniazid**	0.2 (low) / 1.0 (high)	Same
**Ethambutol**	7.5	Same
**Ofloxacin**	2.0	Not set
**Moxifloxacin**	0.5	Same
**Kanamycin**	5.0	Not set
**Bedaquiline**	0.25	Same
**Clofazimine**	0.25	Not set
**Capreomycin**	10.0	Not set
**Ethionamide**	5.0	10.0
**p-aminosalicylic acid**	2.0	Not set

Supplementary Methods 2: Method for DNA extraction

Sputum samples were decontaminated, homogenised and inoculated into mycobacterial growth indicator tubes (MGIT) and incubated in a BACTEC MGIT 960 (BD, NJ, USA) until flagging positive. Positive MGIT tubes were centrifuged at 16,000g for 15 minutes and the supernatant removed. Cells were resuspended in phosphate-buffered saline before undergoing heat killing at 95°C for 1 hour followed by centrifugation at 16,000g for 15 minutes. The supernatant was removed and the sample resuspended in 1mL sterile saline (0.9% w/v). Centrifugation, removal of supernatant and resuspension in 1mL sterile saline was repeated. DNA was extracted with mechanical ribolysis before purification with AMPure XP beads (Beckman Coulter, IN, USA). DNA concentrations were measured with Qubit (ThermoFisher, MA, USA). NEBNext Ultra II DNA (New England Biolabs,

MA, USA) was used for DNA library preparation. Batches of 48 multiplexed samples were sequenced on NextSeq 500 (Illumina, CA, USA) 300-cycle paired end runs with a mid-output kit.

Supplementary Methods 3: Genetic mutations conferring drug resistance

First line drugs (rifampicin, isoniazid, pyrazinamide, ethambutol)	Walker et al (2)
Second line drugs (all others)	Phelan et al (3)

The following additional mutations were included:

**Gene**	**Drug**	**Variant**
atpE	Bedaquiline	D28A
atpE	Bedaquiline	D28G
atpE	Bedaquiline	D28N
atpE	Bedaquiline	D28P
atpE	Bedaquiline	D28V
atpE	Bedaquiline	L59V
atpE	Bedaquiline	E61D
atpE	Bedaquiline	A63P
atpE	Bedaquiline	A63V
atpE	Bedaquiline	I66M
Rv0678	Bedaquiline/Clofazimine	any non-synonymous/frameshift - evaluated individually
pepQ	Bedaquiline/Clofazimine	A14fs
pepQ	Bedaquiline/Clofazimine	L44P
pepQ	Bedaquiline/Clofazimine	R271fs
Rv1979c	Clofazimine	V52G
Rv1979c	Clofazimine	V351A
fgd1	Delaminid	G49fs
fbiA	Delaminid	D49Y
ddn	Delaminid	G81D
ddn	Delaminid	G81S
rplC	Linezolid	T460C
rrl	Linezolid	c1921t
rrl	Linezolid	g2061t
rrl	Linezolid	g2270c/t
rrl	Linezolid	g2294a
rrl	Linezolid	g2299t
rrl	Linezolid	g2447t
rrl	Linezolid	g2576t

rrl	Linezolid	g2576t
rrl	Linezolid	a2689t
rrl	Linezolid	g2746a
rrl	Linezolid	a2810c/t
rrl	Linezolid	g2814t
rrl	Linezolid	c2848a

Supplementary Tables

Supplementary Table 1. Genomic regions covered by resistance bait set for deep sequencing. Resistance conferred lists whether the genomic region is proposed to confer resistance to one or more specific drugs, or is an efflux pump that has the potential to be involved in resistance to multiple drugs. Some variable and housekeeping genes identified from previous studies are also included.

**NC_000962.3 coordinate**		**Description**	**Resistance conferred**
**Start**	**End**		
5139	9918	*gyrB...gyrA*	Fluoroquinolones
53562	55799	*ponA1*	Rifampicin
125542	130641	*ctpI*	Variable gene
222188	223630	*Rv0191*	Efflux pump
244383	247418	*mmpl3*	mmpL3 inhibitors
409261	414338	*iniB…iniC*	Isoniazid
490682	491893	*fgd1*	Delamanid
575247	576890	*mshA*	Ethionamide
629939	631786	*Rv0538*	Variable gene
650678	651604	*Rv0560c*	DprE1 inhibitors
759706	767420	*rpoB…rpoC*	Rifampicin
775485	779587	*mmpL5…Rv0678*	Bedaquiline/Clofazimine
781459	782034	*rpsL*	Streptomycin
800708	802233	*rplC…rplD*	Linezolid
814227	815090	*rpsE*	Unknown
840846	841756	*vapB31…vapC31*	Variable genes
945955	947415	*Rv0849*	Streptomycin/Kanamycin
1010035	1011834	*Rv0907*	Variable gene
1302830	1305601	*fbiC*	Delamanid
1313624	1315291	*pks3*	Variable gene
1361697	1362833	*Rv1218c*	Streptomycin/Kanamycin
1405980	1407440	*Rv1258c*	Streptomycin/Kanamycin
1416080	1417447	*embR*	Ethambutol
1460944	1461390	*atpE*	Bedaquiline
1471745	1476895	*rrs…rrl*	Injectables
1480793	1482601	*Rv1319c*	Variable gene
1586109	1587866	*Rv1410c*	Efflux pump
1624353	1625465	*opcA*	Variable gene
1673299	1675111	*fabG1_promoter*	Isoniazid/Ethionamide
1833441	1835087	*rpsA*	Pyrazinamide
1893476	1894330	*Rv1667c*	Efflux pump

1917839	1918846	*tlyA*	Capreomycin
1929685	1931556	*cycA*	Cycloserine
2101550	2103142	*ndh*	Clofazamine/Ethionamide
2153788	2156211	*katG*	Isoniazid
2156048	2156692	*furA*	Isoniazid
2221618	2223264	*Rv1979c*	Clofazamine
2231579	2232319	*erm(37)*	Macrolides
2288580	2289341	*pncA*	Pyrazinamide
2294430	2307086	*pks12*	Variable gene
2338608	2340974	*Rv2082*	Variable gene
2414833	2416494	*murD*	Variable gene
2434536	2434847	*Rv2172c-idsA2*	Capreomycin
2518014	2519465	*kasA*	Isoniazid
2714023	2715432	*eis*	Kanamycin
2725470	2726880	*oxyR…ahpC*	Isoniazid
2733129	2734244	*rbsK*	Variable gene
2746034	2747698	*folC*	PAS
2859199	2860518	*pepQ*	Bedaquiline
2866367	2867886	*lppA…lppB*	Variable genes
2881308	2882247	*Rv2561*	Variable genes
2986738	2987715	*ribD*	PAS
3004644	3005750	*Rv2688c*	Efflux pump
3017734	3019521	*sigA*	Housekeeping gene
3022360	3023532	*sigB*	Housekeeping gene
3067945	3068188	*thyX-hsdS.1*	PAS
3073579	3074571	*thyA*	PAS
3086719	3088035	*ald*	Cycloserine
3129243	3131873	*Rv2823c*	Variable gene
3152938	3154731	*efpA*	Efflux pump
3196330	3196950	*Rv2887*	DprE1 inhibitors
3245344	3251175	*ppsA*	Variable gene
3272113	3273309	*drrA*	Efflux pump
3336695	3338017	*ddlA*	Cycloserine
3366543	3367367	*Rv3008*	Efflux pump
3430286	3430810	*mmr*	Streptomycin/Kanamycin
3448403	3450091	*Rv3083*	Ethionamide
3511317	3511681	*PPE52-nuoA*	Unknown
3568300	3568779	*whiB7*	Streptomycin/Kanamycin
3624809	3626713	*mtrB*	Variable gene
3640442	3642981	*fbiA…fbiB*	Delamanid
3840093	3841520	*alr*	Cycloserine
3986743	3987399	*ddn*	Delamanid

4007230	4008282	*nat*	PAS
4043761	4044381	*panD*	Pyrazinamide
4155639	4156829	*dnaQ*	Variable gene
4201793	4202713	*proZ*	Efflux pump
4235678	4237264	*dprE1*	DprE1 inhibitors
4239762	4249910	*embC…embB*	Ethambutol
4268824	4269933	*ubiA*	Ethambutol
4325903	4328299	*ethA…ethR*	Ethionamide
4407427	4408302	*gid*	Streptomycin

Supplementary Table 2. Associations between mixed infection and patient and bacterial factors.

Characteristic	Number (%)	p-value (no. in category)
**PATIENT FACTORS**		
**Age Category**		
<25	1/32 (3.03%)	0.195
25-30	5/68 (7.35%)	N=346
30-35	8/71 (11.27%)	
35-40	4/54 (7.41%)	
40-50	1/73 (1.37%)	
≥50	2/47 (4.26%)	
**Sex**		
Male	15/203 (7.39%)	0.221
Female	6/143 (4.20%)	N=346
**Previous TB**		
No	11/178 (6.18%)	0.963
Yes	10/165 (6.06%)	N=343
**HIV status**		
No	6/66 (9.09%)	0.244
Yes	15/283 (5.30%)	N=349
**CD4 count**		
≥200	6/96 (6.25%)	0.103
<200	2/109 (1.83%)	N=205
**Taking antiretroviral therapy**		
No	5/104 (4.81%)	0.983
Yes	9/185 (4.86%)	N=289
**Cavitation on chest radiography**		
No	4/93 (4.30%)	0.544
Yes	2/28 (7.14%)	N=121
**On TB treatment at enrolment**		
No	11/164 (6.71%)	0.656
Yes	10/180 (5.56%)	N=344
**BACTERIAL FACTORS**		
**DR profile**		
DS	6/89 (6.74%)	0.456
MDR/RR	12/192 (6.77%)	N=352
Pre-XDR/XDR	2/71 (2.82%)	

Supplementary Table 3. Correlates of month six outcome showing hazard ratio and 95% confidence intervals (95% CI) and p-values calculated by logistic regression. All hazard ratios refer to patients with single strain infection, except for the hazard ratio for mixed infection.

**Characteristic**	**Hazard ratio (95% CI)**	**p-value**
**PATIENT FACTORS**		
**Age (per year increment)**	1.02 (0.99-1.04)	0.2561
**Male sex**	1.97 (1.11-3.48)	0.0189
**Positive HIV status**	3.78 (1.31-10.94)	0.0043
**CD4 count ≥200**	0.56 (0.27-1.18)	0.1278
**Taking antiretroviral therapy**	2.55 (1.21-5.38)	0.0090
**Previous TB**	1.41 (0.80-2.50)	0.2331
**Cavitation on chest radiography**	0.63 (0.17-2.39)	0.4859
**On TB treatment at enrolment**	0.51 (0.29-0.90)	0.0184
**BACTERIAL FACTORS**		
**Mtb lineage 2 (v lineage 4)**	0.98 (0.54-1.79)	0.9602
**Drug resistance profile (v DS)**		
**MDR/RR**	1.62 (0.77-3.42)	0.204
**Pre-XDR/XDR**	2.42 (1.04-5.59)	0.039
**Mixed infection**	1.28 (0.45-3.69)	0.6415

Supplementary Table 4. Classification, description and nucleotide diversity (π) of the 10 most diverse coding genes in baseline sequences.

**Gene**	**Classification**	**Functional category**	**Functional description**	**π**
Rv1319c	Non-Essential	intermediary metabolism and respiration	Probable adenylate cyclase	2.65×10^{-4}
Rv3424c	Non-Essential	conserved hypotheticals	Unknown	2.55×10^{-4}
Rv2825c	Non-Essential	conserved hypotheticals	Unknown	1.51×10^{-4}
Rv1435c	Non-Essential	cell wall and cell processes	Unknown	1.07×10^{-4}
Rv2319c	Essential	virulence, detoxification, adaptation	Unknown	9.26×10^{-5}
cysA2	Non-Essential	intermediary metabolism and respiration	Sulfotransferase	7.41×10^{-5}
fadD18	Non-Essential	lipid metabolism	Lipid degradation	7.39×10^{-5}
Rv2081c	Non-Essential	cell wall and cell processes	Unknown	6.46×10^{-5}
Rv0740	Non-Essential	conserved hypotheticals	Unknown	5.82×10^{-5}
Rv1765c	Antigen	conserved hypotheticals	Unknown	5.32×10^{-5}

Supplementary Table 5. Frequencies and outcomes of baseline hetRAVs.

Patient ID	Drug	Mutation	Allele frequency				
			Sample 1	Sample 2	Sample 3	Sample 4	Sample 5
K0045	Rifampicin	Asp435Val	64.1%	42.6%			
K0045	Rifampicin	Ser450Leu	31.3%	56.1%			
K0056	Rifampicin	Leu430Pro	15.4%	77.8%	100.0%		
K0056	Rifampicin	Asp435Val	75.7%	18.4%	0.0%		
K0062	Rifampicin	Ser450Leu	9.1%	0.0%	0.0%		
K0084	Rifampicin	His445Tyr	7.8%	0.0%	0.0%		
K0084	Rifampicin	Ser450Leu	92.3%	100.0%	100.0%		
K0085	Rifampicin	His445Asp	88.6%	100.0%	100.0%		
K0085	Rifampicin	Leu452Pro	9.1%	0.0%	0.0%		
P0117	Rifampicin	His445Tyr	41.4%	0.0%	100.0%		
P0117	Rifampicin	His445Arg	49.2%	98.0%	0.0%		
P3418	Isoniazid (katG)	Gly297Val	48.5%	11.5%			
K0049	Pyrazinamide	Thr153fs	92.7%	96.8%	92.1%		
P0037	Pyrazinamide	Thr153fs	91.3%	100.0%			
P0044	Pyrazinamide	Thr153fs	93.1%	97.2%	94.1%		
P3505	Pyrazinamide	Thr153fs	93.6%	96.4%			
P3519	Pyrazinamide	Thr153fs	94.8%	93.3%			
P3535	Pyrazinamide	Thr153fs	93.3%	97.0%			
K0006	Ethambutol	Asp328Tyr	43.9%	0.0%	0.0%		
P0027	Fluoroquinolones	Glu501Asp	30.0%	14.0%	5.6%	61.7%	51.7%
P0027	Fluoroquinolones	Ala90Val	39.1%	36.5%	91.3%	10.7%	0.0%
P0027	Fluoroquinolones	Asp94Gly	28.4%	42.9%	0.0%	0.0%	33.9%
P0117	Fluoroquinolones	Asn499Tyr	33.9%	0.0%	100.0%		
P0117	Fluoroquinolones	Ala90Val	14.7%	0.0%	0.0%		
P0117	Fluoroquinolones	Asp94Gly	47.9%	100.0%	0.0%		
P0040	Second line injectables	Ala67fs	88.2%	94.4%			
P0027	Bedaquiline	Arg109Leu	75.4%	92.3%	94.3%	22.5%	31.3%
P0027	Bedaquiline	Arg156fs	12.9%	7.8%	8.1%	54.1%	30.2%
P0150	Bedaquiline	Glu49fs	72.1%	96.1%	96.9%		
P3427	Bedaquiline	Met146Thr	2.0%	0.0%			

Supplementary Figures

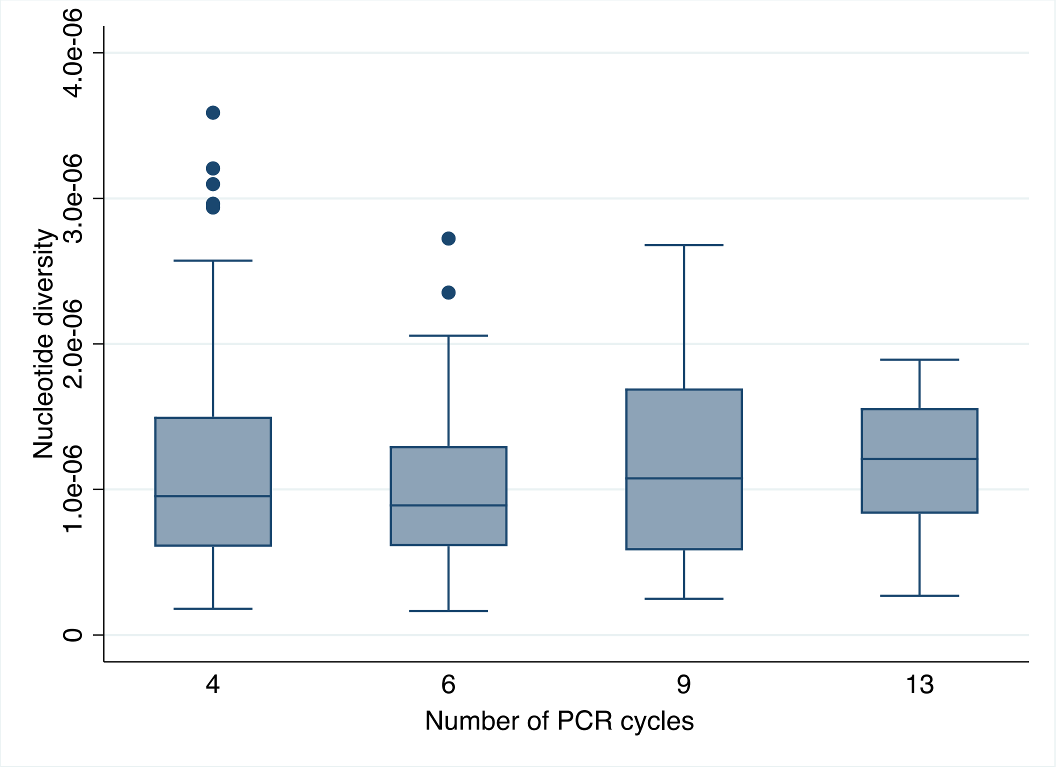

Supplementary Figure 1. Nucleotide diversity median and interquartile range stratified by number of PCR cycles during library preparation. Outlying values are marked with circles. We observed no statistically significant difference between any of the four classes (equality of medians test).

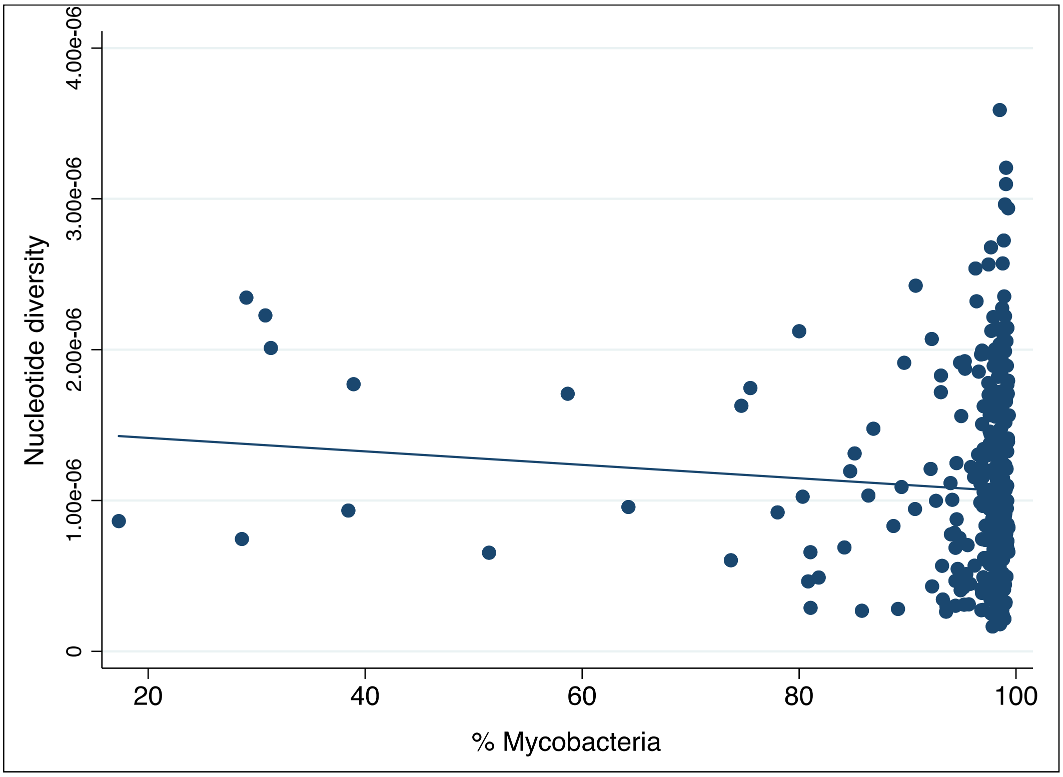

Supplementary Figure 2. Scatter plot and linear regression line of proportion of *k*-mers assigned to *Mycobacteria* by Kraken2 and nucleotide diversity. R-squared 0.6%, p=0.16 for linear association using linear regression.

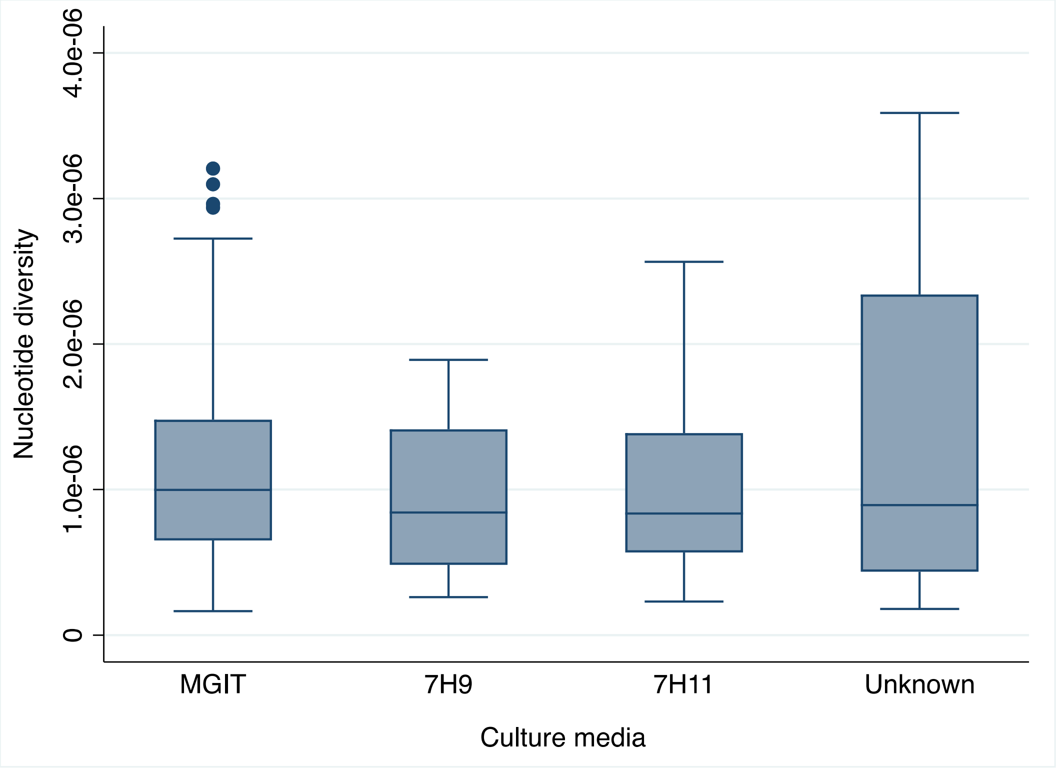

Supplementary Figure 3. Nucleotide diversity stratified by sample culture media: MGIT (initial culture), 7H9 (single subculture), 7H11 (solid plate) or unknown. We observed no statistically significant difference between any of the culture media (Wilcoxon rank-sum test).

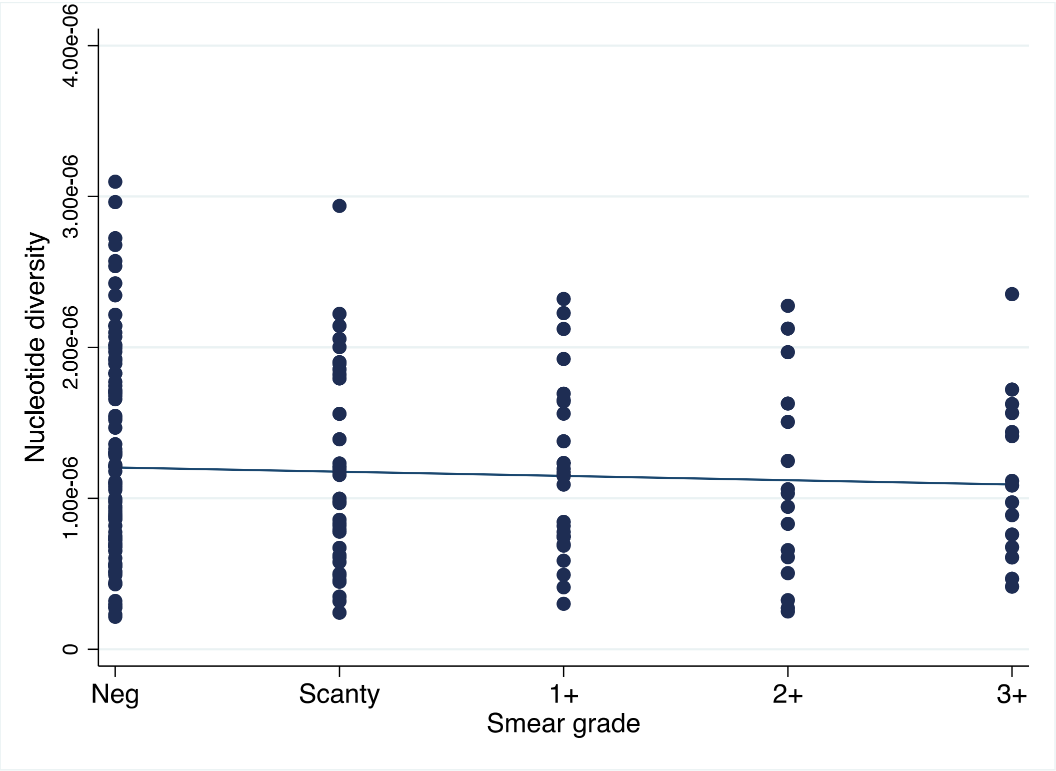

Supplementary Figure 4. Linear regression of increasing smear grade level against nucleotide diversity with fitted regression line. R-squared 0.3%, p=0.43 for linear association using linear regression.

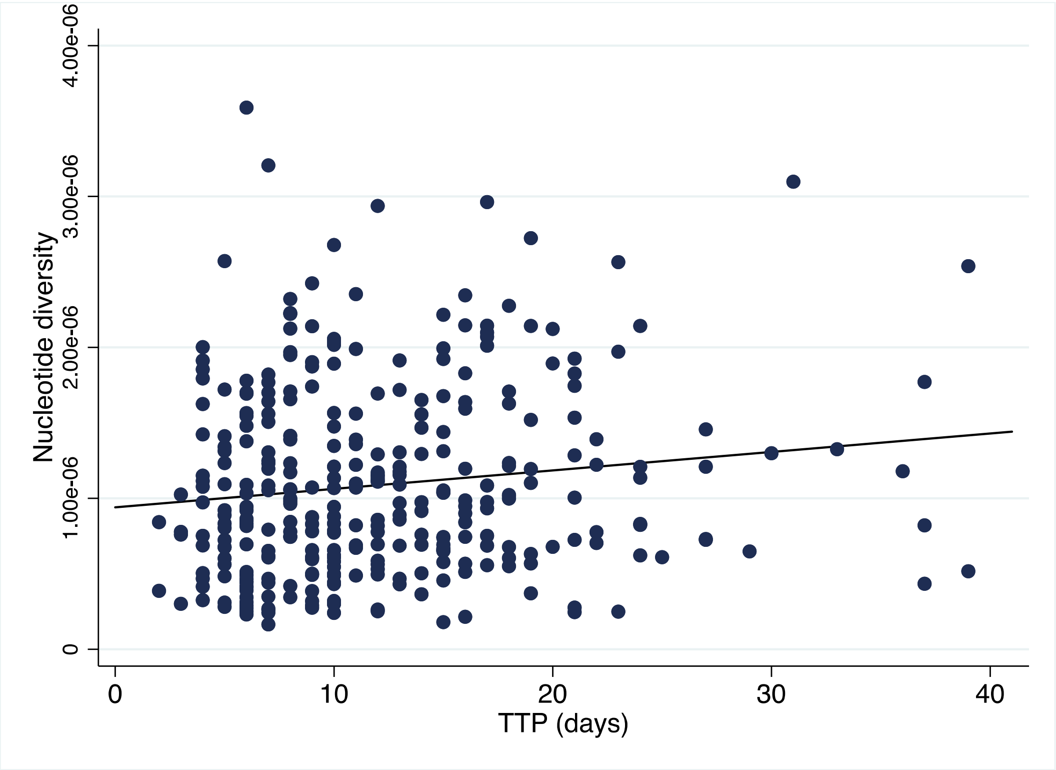

Supplementary Figure 5. Association between increasing MGIT time to positivity (TTP) and nucleotide diversity with fitted linear regression. R-squared 1.8%, p=0.017 for linear association using linear regression.

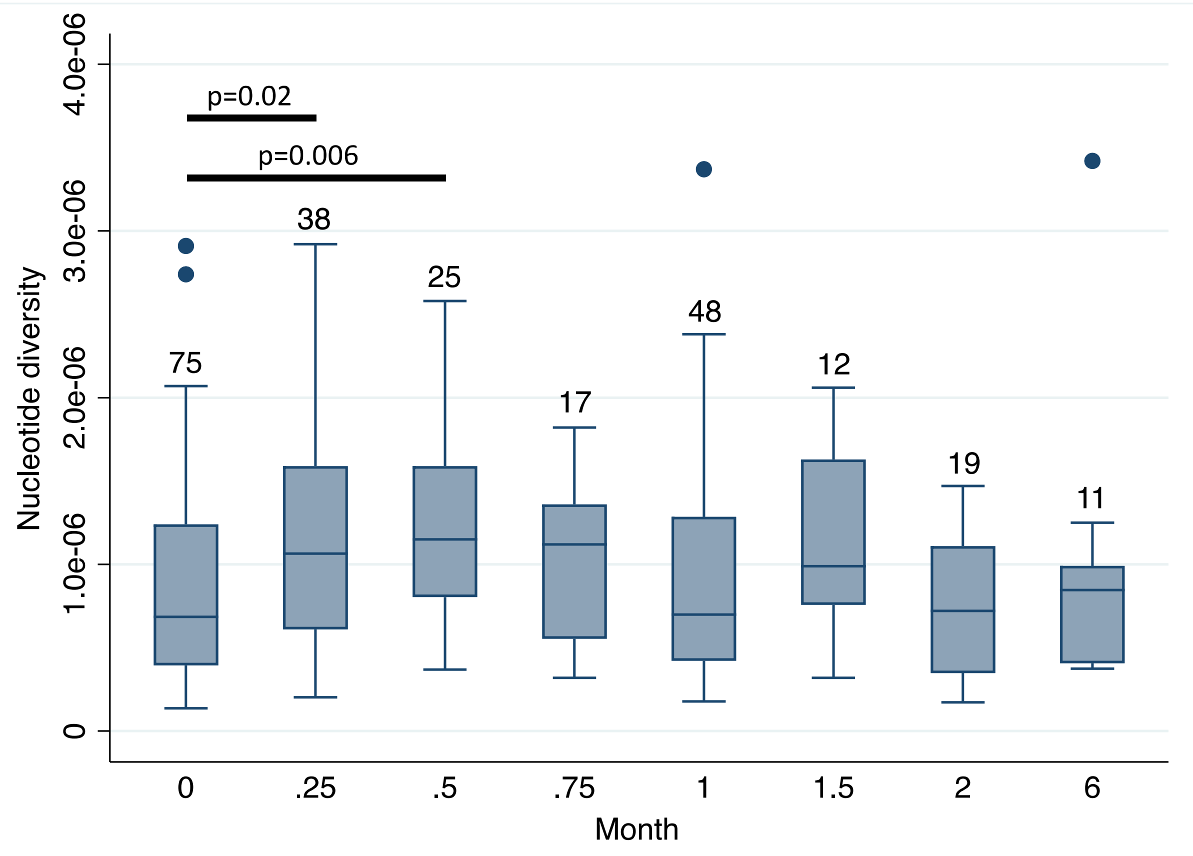

Supplementary Figure 6. Box plot showing median and interquartile range of nucleotide diversity in sequential samples from patients who had at least one positive culture after week 2 at timepoints where there were ≥5 samples. Dots mark outlying values. Numbers indicate number of isolates at each timepoint. Bars indicate where distributions are statistically significantly different from baseline, with p-values calculated by Wilcoxon rank-sum test.

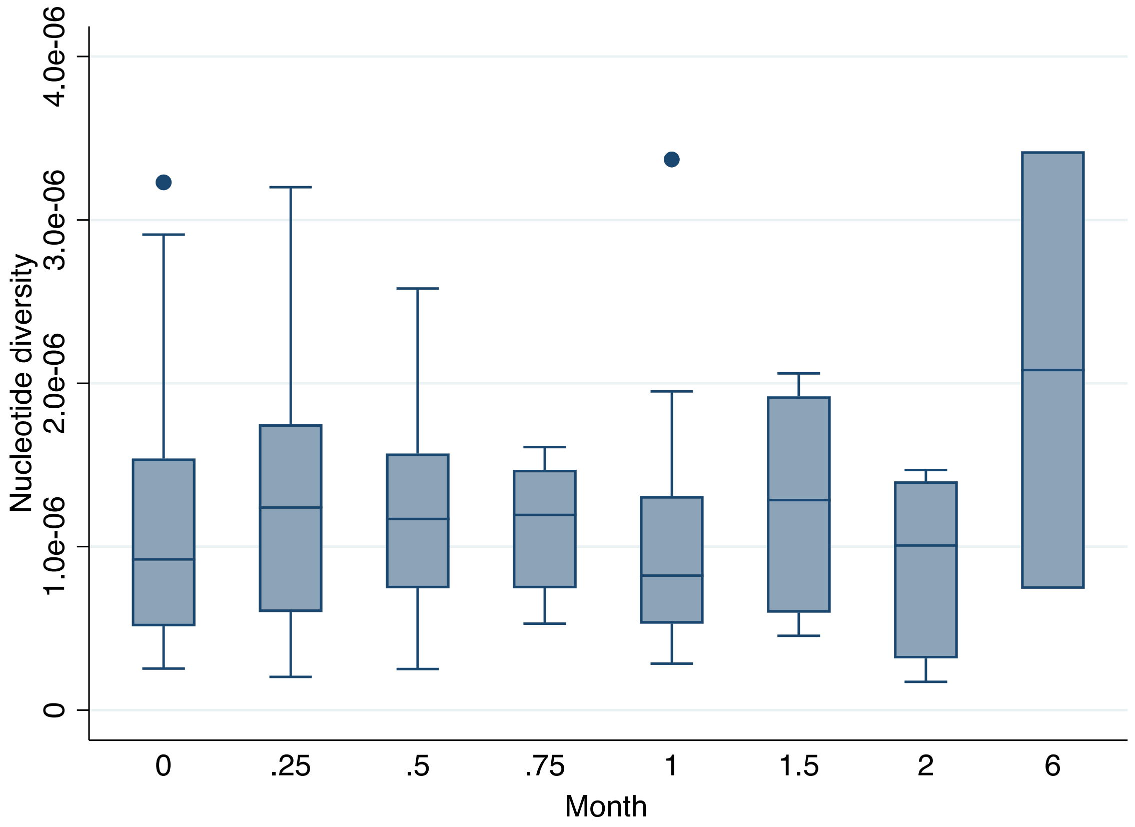

Supplementary Figure 7. Box plot showing median and interquartile range of nucleotide diversity in sequential isolates from patients who were not receiving any TB treatment at the time of enrolment. There were no statistically significant differences between timepoints (Wilcoxon rank-sum test).

References

1. World Health Organization. Technical report on critical concentrations for TB drug susceptibility testing of medicines used in the treatment of drug-resistant TB. Geneva; 2018.
2. Prediction of Susceptibility to First-Line Tuberculosis Drugs by DNA Sequencing. N Engl J Med. 2018 Sep 26;379(15):1403–15.
3. Phelan JE, O'Sullivan DM, Machado D, Ramos J, Oppong YEA, Campino S, et al. Integrating informatics tools and portable sequencing technology for rapid detection of resistance to anti-tuberculous drugs. Genome Med. 2019;11(1):41.
